# Prevalence of depression and its associated sociodemographic factors among Iranian female adolescents in secondary schools

**DOI:** 10.1186/s40359-019-0298-8

**Published:** 2019-04-24

**Authors:** Babak Moeini, Saeed Bashirian, Ali Reza Soltanian, Ali Ghaleiha, Malihe Taheri

**Affiliations:** 1 Social Determinants of Health Research Center, Hamadan University of Medical Sciences, Hamadan, Iran; 2 Department of Public Health, School of Public Health & Social Determinants of Health Research Center, Hamadan University of Medical Sciences, Hamadan, Iran; 3 Modeling of Noncommunicable Diseases Research Center, School of Public Health, Hamadan University of Medical Sciences, Hamadan, Iran; 4 Research Center for Behavioral Disorders and Substances Abuse, Hamadan University of Medical Sciences, Hamadan, Iran; 5 Department of Public Health, School of Public Health, Hamadan University of Medical Sciences, Hamadan, Iran

**Keywords:** Adolescent depression, School girls, Prevalence

## Abstract

**Background:**

Across the globe, depression is a common psychiatric disorder and is the main cause of disability among adolescents. To this end, this study was conducted to screen for the prevalence of depression among secondary school female students in the city of Hamadan, in western Iran.

**Methods:**

In this cross-sectional study, a total of 670 secondary school female students, within the age range of 15–18 years were investigated using multistage random sampling method. Moreover, the Persian version of Center for Epidemiologic Studies Depression Scale (CES-D) and a researcher-designed questionnaire containing demographic variables were employed as research instruments. Analyses of the findings were made using SPSS version 16 software followed by stratified logistic regression model, which was performed for correlation analysis.

**Results:**

The mean (standard deviation) age of students was 16.2 (0.68) years. The prevalence of severe depression in female students estimated by the Center for Epidemiologic Studies Depression Scale (CES-D) was equal to 52.6%. A statistically significant relationship was also observed to exist between prevalence of depression and type of school (*P* < 0.001), family income (P < 0.001), living in the suburbs (*P* < 0.001), and field of study at school (P < 0.001). However, no statistically significant correlation was found between depression among students and school grade, type of living with parents, father’s education and occupation, mother’s education and occupation, and family size.

**Conclusion:**

Depression was prevalent among the secondary school female students examined and it significantly correlated with socioeconomic status. Therefore, periodic screening, psychological training programs, proper diagnosis of high-risk individuals in secondary schools, and early intervention among secondary school female students are urgently needed.

## Background

Adolescence can be described as a transitional period from childhood to adulthood, which begins with puberty and involves profound transformations in social, physical and psychological aspects [1] that could be stressful for the adolescent, and such stress may render them feeling confused, negative and also depressed. In this regard, several studies have reported an increased prevalence rate of adolescent depression in the range of 10–20% [2].

Prior studies revealed that sociodemographic factors such as older age, parents’ occupational status, marginalization [3], female gender [4], lower education levels of parents and living conditions with parents [5] were important risk factors for depression among adolescents. In addition, psychosocial risk factors for depression are family disputes, low socioeconomic status, and undesirable academic performance [6].

As mentioned, it has been reported that girls can experience higher rates of depression compared with boys in adolescence, perhaps due to different biological, psychological, family upbringing and socio-cultural factors [7]. A wide range of possible psychosocial risk factors for depression in girls varies considerably in puberty, such that the ratio of female depression is around 1.7 to 2 with the onset of puberty, and 2–3 times across adulthood [8]. Therefore, female depression is a public health priority.

Depression is a serious mental disorder among adolescents, which can often have an impact on social functioning, family relationships, and academic performance in adolescents [9]. These problems can become chronic, leading to mental and substance use disorders which is the cause of about 40·5% of disability adjusted life years (DALYs) in adolescents [10]. In worst cases, depression can lead to suicide [11]. Despite its serious consequences, depression in adolescent generally remains under-diagnosed and under-treated [12].

In Iran, as a developing country, adolescents constitute about one-third of the population [13] and the given studies confirm the prevalence of serious psychological problems of depression and stress among Iranian adolescents which varies between 14.77 to 72% [14]. Also according to previous studies, the years of disability due to depression in Iran are higher compared with other developing countries [15]. For this reason, proper recognition of adolescent depression, its associated risk factors, combined with early intervention as well as best treatment, can characterize preventive strategy as being potentially significant and cost-effective, particularly in developing countries such as Iran.

Prior studies in Iran have focused on men adolescence [11], both gender [12], in highly vulnerable adolescence such as after the earthquake disaster [13] and adolescents living with deficiency or illness [14]. Additionally, Iranian girls have much more limitations than boys. Also, a greater control and limitation is imposed by the society and families on girls’ behaviors and life compared to boys. One possible cause of depression can be perceived limitations in the personal and social life of girls. This problem was likely more complex and severe in girls than boys [16]. However, most female adolescents with depression are not diagnosed as a result of such restrictions [17]. Therefore, in the present study, an attempt was made to understand prevalence and sociodemographic factors influencing depression in female adolescents in west of Iran.

The first objective in this study was to estimate the prevalence of mild depression, moderate and severe depression among female adolescents aged 14–18 years in Hamadan, Iran. The second and third objectives were to evaluate the possible relationship between female’s depression and socio-demographics factors including individual characteristics and family characteristics in the study group.

Our hypotheses were as follows: 1) based on other studies conducted in Iran, the prevalence of depression among the target group in present study would be high (over 50%) [14]. 2) Based on the findings of other studies [18–20], we assume that, there would be a relationship between the incidence of depression and the individual characteristics of female students, including the school grade, field of study, and the type of school. 3) Based on other studies [21–24], it is assumed that there would be a relationship between the incidence of depression and the family characteristics of female students, such as family income, parental education, and parents’ employment status, place of residence and living conditions with parents.

### Theoretical framework

As noted in the study by Meredith et al. [25] based on theoretical framework of the Social Production Function Theory [26] humans attaining their ultimate goal of ‘psychological wellbeing’. At the lowest level, social, economic and cultural resources are important for ‘psychological wellbeing’. If consider depressive symptoms as an outcome from an absence of psychological wellbeing, effects of (lower levels of) resources based on these theoretical concepts might be a suitable theoretical approach to describe variances in socio-demographic associated to depressive symptoms. Accordingly, with a proposition that a lower level of resources might persuade depression. Systematically elaborated on this rather general proposition by formulating more specific propositions that were based on previous research findings [27].

## Method

### Study design and sample

The study population of this cross-sectional research consisted of secondary school female students in the city of Hamadan, west of Iran. Collection of data was from 15th of April to 15th of June 2016. The education system in Iran is such that schools are separate by gender, and there are separate schools for boys and girls from pre-elementary schools to the end of secondary school.

According to previous studies [28], the prevalence of mild depression was reported to be 20%. Therefore, given the formula of (z^2^ 1-α/2) σ^2^ /d^2^, 95% confidence level, a maximum significant difference of 0.04 and the nonresponse rate of 20%, the sample size was estimated to be 673 students.

Cluster multistage sampling method was performed for selection of the study sample. To attain this purpose; first, the list of female secondary school students in the city of Hamadan was prepared based on the information provided by the Education Office of Hamadan.

Subsequently, the female secondary school students of the dual administrative districts of the Education Office of Hamadan were separated into two groups depending on access to healthcare services, as advantaged (downtown areas) and non-advantaged (the suburbs).

**Fig. 1:**
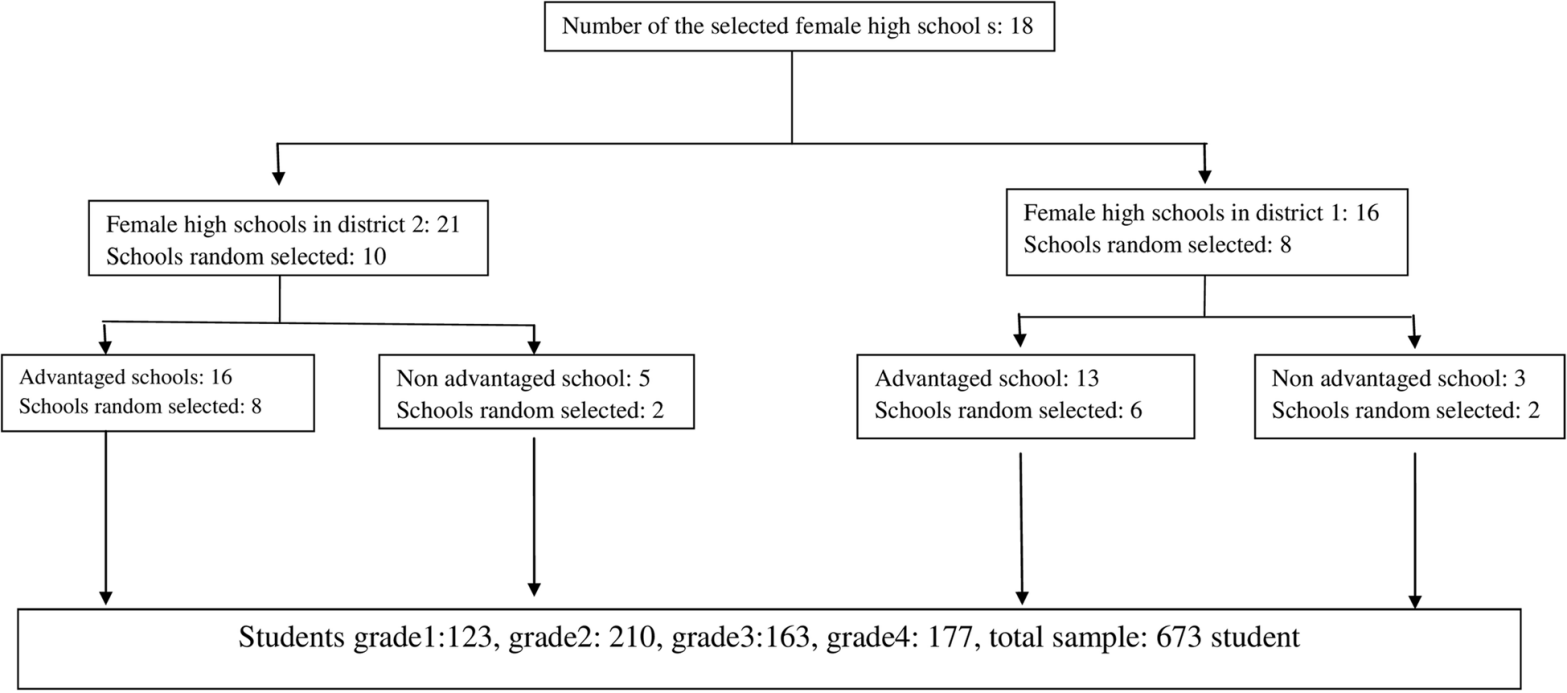
Flow diagram illustration of the sampling process and selection of study subjects from the two general educational districts that 23 schools were finally chosen, and each school four classes were selected randomly

The number of female secondary school students that signed up from each district was proportional to the number of secondary schools for girls and the number of the students at each school grade within that district. In district 1, 32 classes in 8 schools (50% of eligible classes, *n* = 65) participated using simple random sampling method. In district 2, 40 classes in 10 schools participated (50% of eligible classes, *n* = 80) using simple random sampling method. In district 1, 8 classes participated in grade 9 (14 to 15 years old), 8 classes in grade 10 (15 to 16 year olds), 9 classes in grade 11 (16 to 17 years old) and 7 classes in pre-university grade (17 to 18 years old). In district 2, 10 classes participated in grade 9 (14 to 15 years old), 9 classes in grade 10 (15 to 16 years old), 10 classes in grade 11 (16 to 17 years old) and 11 classes in pre-university grade (17–18 years old). Therefore, a total of 720 students were identified; from which 24 students and 15 parents withdrew from the study, 3 students were above 18 years, 2 students were under psychiatric medications, and 3 students suffered from chronic diseases, and were all excluded from the present study. This gave a final total of 673 students who participated in this study (Fig. 1).

The inclusion criteria in this study were female secondary and pre-university school students aged 14–18 years, with no chronic diseases, taking no psychiatric medications and having informed written consent from parents / guardians. Information was provided to adolescents to seek consent from their parents at home, after which parents that gave their children consent to attend the study signed the consent forms.

For completion of all questionnaires by the selected students, apart from the day of the survey, three days of follow-up were carried out by the researcher at the schools to ensure that there are no missing data. No questionnaires were removed. In addition, all questionnaires were completed during regular school hours by the students under the supervision of the researcher (corresponding author) and this took approximately an hour to complete. All questionnaires were anonymous and provided in Persian language; the official language used in Hamadan (West of Iran).

The questionnaires were filled out in the classroom without the presence of teachers and other school staff to maintain data confidentiality. For anonymity, the student’s names were not recorded on the questionnaires, but only the third last digits of each student’s code was used for identification.

### Measurement scales

#### Socio-demographic variables

First, the adolescents completed a researcher-designed questionnaire which included items about age, school grade, type of school, and some items about family status including family size, family income, living conditions with parents, mother’s occupation, father’s occupation, mother’s level of education, and father’ level of education. It needs to be explained that, given the laws of the Iranian education system, asking questions about some of factors associated with depression from school students have legal restriction. Questions such as alcohol use, smoking, drug use, having sex, suicidal thoughts and religion, so these factors were removed from the questionnaire.

#### Depression symptoms

Depression symptoms were assessed using the self-administered Persian version of Center for Epidemiologic Studies Depression Scale (CES-D). In this study, the full 20-item version was used. Each items were scored from 0 to 3 on the basis of ‘how often have you felt this way during the past week’, 0 - rarely or none of the time (less than 1 day), 1 - some or a little of the time (1–2 days), 2 - occasionally or a moderate amount of time (3–4 days), and 3 - most or all of the time (5–7 days). It should be noted that negative statements as 4, 8, 12, and 16 were recoded.

The items contained declarations about depressive mood, reduced appetite, sleep disorder, feeling of worthlessness and hopelessness and loss of concentration [29]. Total score for this research instrument was between 0 and 60. Higher scores meant higher level of depression. According to this research instrument, depression scores lower than 15 were considered normal and meant no depression, scores from 15 to 21 implied mild to moderate depression, and scores higher than 22 implied severe depression. The validity and the reliability of Persian version of this questionnaire were examined by Amiri et al. [30] in Iran. To assess the reliability of this Persian version; test-retest, split-half, and internal consistency methods were employed. The reliability values within 2 weeks were 0.77, 0.92, and 0.85 for the test-retest, split-half, and internal consistency using Cronbach’s alpha methods, respectively. To determine the validity of the questionnaire, convergent validity was used, so that the correlation coefficient of the scores of the CES-D for 95 subjects was assessed using Beck Depression Inventory. A value of 0.65 was obtained as the correlation value between the two tests which was significant at the level 0.01. In this study, an internal consistency of 0.87 was obtained for the CES-D scale.

### Statistical analysis

Statistical analysis was carried out using SPSS, version 16. Frequencies and percentages were used to obtain the prevalence and general characteristics of the participants. Multiple logistic regression used to determine the relationship between demographic variables and depression symptoms in the participants. Statistical significance was less than 0.05.

## Result

### Sample characteristics

Table 1 presents the detailed baseline characteristics of the study participants. The mean age of the students was 16.2 years (SD = 0.68). Sixty-eight percent of the students were from families with income less than $7500 per year, and 82% lived with both parents.

**Table 1:** Baseline information on the study sample

	Schoolgrade				Field of study					Type of school		Place of residence	
	1	2	3	Pre-university	Mathematics	Experimental Sciences	Humanities	KVD ^*^	Technical	public	private	downtown areas	suburbs
Study population	123	210	163	177	73	228	206	68	96	513	160	352	321
Family income													
below 500 thousand tomans (about 130 dollars)	4	3	2	2	1	6	2	0	2	8	3	6	5
500 thousand-1.5 million tomans (130–375 dollars)	30	58	63	58	24	72	66	17	30	164	45	108	101
1.5–2.5 million tomans (375–625 dollars)	42	71	48	78	26	74	75	30	32	176	63	126	113
above 2.5 million tomans (> 625 dollars)	47	78	50	39	22	76	63	21	32	165	49	112	102
Mother’s education													
< diploma	3	9	10	5	2	10	8	4	3	23	4	19	8
diploma	60	65	41	50	21	68	66	24	26	159	47	96	110
associate’s degree	17	39	46	59	16	59	62	15	19	125	36	84	77
bachelor’s degree	45	66	56	79	26	72	82	20	35	164	52	111	105
master’s degree and PhD	8	11	10	34	8	19	18	5	13	42	21	42	21
Father’s education													
below diploma	12	9	5	13	3	15	13	4	4	27	12	22	17
diploma	21	50	54	34	20	58	45	22	14	125	34	66	73
associate’s degree	22	29	26	37	19	48	30	14	5	85	29	62	52
bachelor’s degree	46	81	45	58	15	68	75	22	48	183	47	106	124
master’s degree and PhD	22	41	33	35	15	41	43	6	27	93	38	76	55
Mother’s occupation													
houswife	75	120	106	115	42	139	130	40	64	324	92	209	207
employed	48	90	57	62	31	89	76	28	32	189	68	143	114
Father’s occupation													
unemployed	19	14	8	10	10	17	15	5	4	16	35	41	10
employed	104	196	155	167	63	211	191	63	92	336	286	472	150
living with parents													
both of them	86	186	139	142	61	189	162	58	79	410	143	292	261
with mother due to divorce	16	13	9	15	3	18	23	2	7	47	6	24	29
with father due to divorce	11	4	9	17	2	12	16	5	6	37	4	21	20
with mother due to father’s death	6	4	6	0	3	6	2	1	4	13	3	10	6
with father due to mother’s death	4	3	0	3	2	3	3	2	0	6	4	5	5
other	0	0	0	0	2	0	0	0	0	0	0	0	0
Family size													
3 members	60	97	81	7	33	111	95	30	44	227	88	174	141
4 members	37	42	37	37	16	45	53	15	24	122	31	79	74
5 members and more	26	71	35	63	24	72	58	23	28	164	41	99	106

KVD* Kar va Danesh (Job and Knowledge: a new major in Iranian high schools)

### Prevalence and factors associated with depression symptoms

**Fig. 2:**
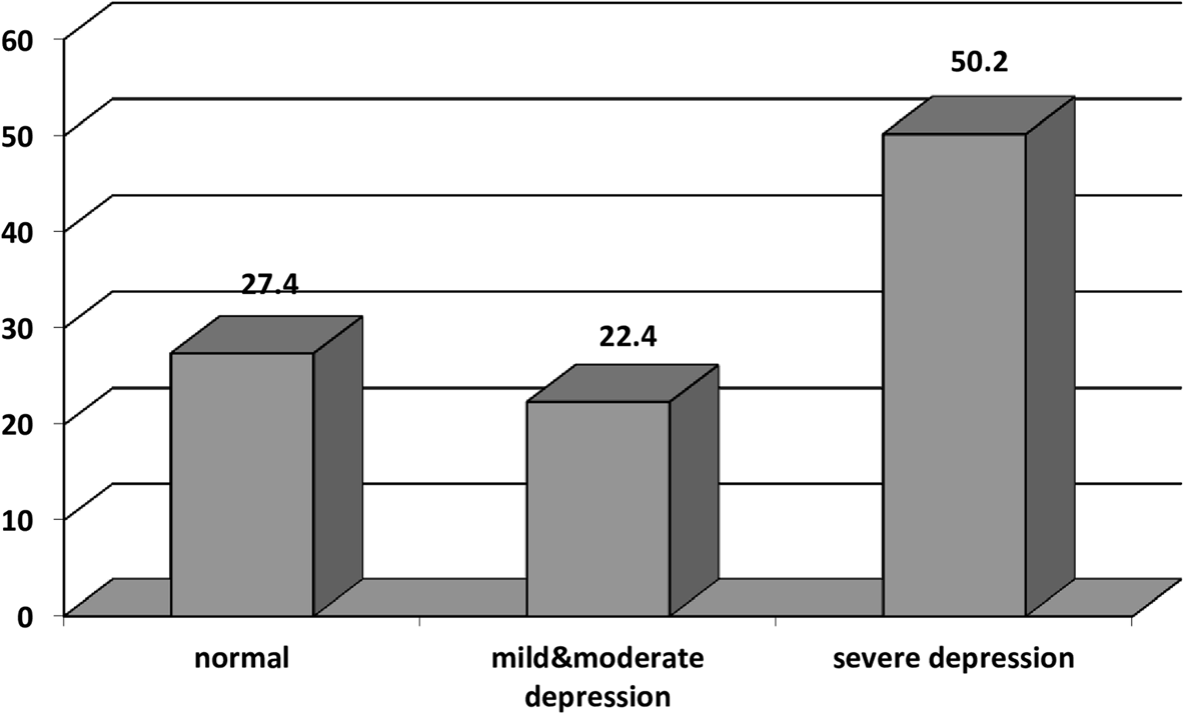
Screening for depression by the CES-D

Table 2 presents the prevalence rate of depression among female students in the city of Hamedan in this study. From the table, about half of the students had severe depression and almost a quarter of them suffered from mild and moderate depression (Fig. 2). The mean score of depression was 22.47 (SD = 12.34).

**Table 2:** Prevalence of depression stratified by target variables

			Depression Levels						
	severe		Mild& Moderate		Normal		Score means		
	n	%	n	%	n	%	Estimate	C195%	*p*-value
Family income ^a^									
below 500 thousand tomans (about 130 dollars)	8	72.7	2	18.1	1	9.1	−1.598	(−2.309,-.887)	.000
500 thousand-1.5 million tomans (130–375 dollars)	111	53.1	36	17.2	62	29.6	−.377	(−.660,-.095)	.009
1.5–2.5 million tomans (375–625 dollars)	124	51.8	41	17.1	74	30.9	−.245	(−526,-.036)	.087
above 2.5 million tomans (> 625 dollars) (Reference category)	115	53.7	39	18.2	60	28.03			
Mother’s education ^b^									
below diploma	18	66.6	3	11.1	6	22.22	−.0225	(−.647,.197)	.296
diploma	95	46.1	45	21.8	66	32.03	−.028	(−.468,.412)	.900
associate’s degree	87	54.03	27	16.7	47	29.1	−.048	(−.486,.390)	.830
bachelor’s degree and higher (Reference category)	158	56.6	43	15.4	78	27.9			
Father’s education ^c^									
below diploma	24	61.5	5	12.8	10	25.6	−.206	(−.528,.115)	.208
diploma	73	45.9	32	20.1	54	33.9	−.100	(−.468,.267)	.593
associate’s degree	60	52.6	20	17.5	34	29.8	.037	(−.287,.360)	.824
bachelor’s degree and higher (Reference category)	201	55.6	61	16.8	99	27.42			
Mother’s occupation ^d^									
housewife	218	52.5	79	19	119	28.6	.048	(−.180,.277)	.678
employed (Reference category)	140	54.4	39	15.1	78	30.3			
Father’s occupation ^e^									
unemployed	29	56.8	6	11.7	16	31.3	.155	(−.116,.425)	.262
employed (Reference category)	329	52.8	112	18	181	29.09			
School grade									
1	66	53.6	23	18.6	34	27.6	−.007	(−.444,.458)	.975
2	108	51.4	37	17.6	65	30.9	−.089	(− 480,.302)	.655
3	85	52.1	36	22	42	25.7	−.073	(−.346,.491)	.734
Pre-university (Reference category)	99	55.9	22	12.4	56	31.6			
Field of study ^f^									
Mathematics	37	50	12	16.2	25	34.2	−1.137	(−1.768,-.506)	.000
Experimental Sciences	108	47.3	39	17.1	81	35.5	−1.236	(−1.755,-.718)	.000
Humanities	96	46.6	39	18.9	71	34.4	−1.230	(−1.755,-.706)	.000
KVD	46	67.6	13	19.1	9	13.23	−.320	(−1.005,.364)	.359
Technical (Reference category)	72	74.2	15	15.4	10	10.3			
Type of school ^g^									
public	288	56.1	80	15.5	145	28.26	.303	(.057,.549)	.016
private (Reference category)	70	43.7	38	23.7	52	32.5			
Place of residence ^h^									
suburbs	165	52.7	52	16.6	96	30.67	.291	(−.014,.580)	.006
downtown areas	193	53.11	66	18.3	101	31.46			
Living with parents ^*i*^									
both of them	289	52.2	99	17.9	165	29.8	.340	(−.114,.795)	.142
with mother due to divorce	26	49.05	9	16.1	18	33.9	.511	(−.034,1.056)	.066
with father due to divorce	26	63.4	5	12.1	10	24.3	.503	(−.293,1.299)	.215
with mother due to father’s death	11	68.7	3	18.7	2	12.5	.382	(−.610,1.373)	.450
with father due to mother’s death (Reference category)	6	60	2	20	2	20			
R^2^	0.41								

*CI* Confidence interval

a. below 130 dollars = 1, 130–375 dollars = 2, 375–625 dollars = 3 and > 625 dollars = 4

b. below diploma = 1, diploma = 2, associate’s degree = 3 and bachelor’s degree and higher = 4

c. below diploma = 1, diploma = 2, associate’s degree = 3 and bachelor’s degree and higher = 4

d. housewife = 1, employed = 2

e. unemployed = 1, employed = 2

f. Mathematics = 1, Experimental Sciences = 2, Humanities = 3, KVD = 4, Technical = 5

g. public = 1, private = 2

h. suburbs = 1, downtown areas = 2

i. both of them = 1, with mother due to divorce = 2, with father due to divorce = 3, with mother due to father’s death = 4 with father due to mother’s death = 5

There was no evidence of association between the parent’s marital status, the level of education and occupation of the parents with students’ depression.

No independent association with depressive symptoms was apparent for school grade level, although the second-grade students had the highest prevalence rates of depression symptoms.

Four risk factors linked to the low income family (95% CI −.526–.36, *p* ≤ 0.05), Kar va Danesh^1^ fields of study (95% CI -1.005 - .364, p ≤ 0.05), living in the suburbs, Studying in public schools (95% CI .514–.580, p ≤ 0.05) increased the risks for symptoms of depression in the subjects (Table 2).

Feeling alone (73%), suffering from crying seizures (55%) and feeling sad (67%) were reported by the greatest number of depressed respondents.

In present study % 42 of the depression variance was explained by the sociodemographic variables (R^2^ = 0.42).

## Discussion

### The prevalence of depressive symptoms of the female students

The present study contributes to the research literature on prevalence of depression symptoms and its associated related factors among female adolescents in Iran. The findings of the present study indicated high prevalence rate of depression (72.6%) in female adolescent in the city of Hamadan, which is in agreement with the studies conducted among Iranian adolescents. As a systematic review study, the prevalence rates of depression in different Iranian populations could vary from 5.69 to 73% [31]. Other studies conducted in Iran have similarly reported high prevalence rates of depression and anxiety disorders among children and adolescents [14, 32]. Moreover, several investigations in other Middle Eastern countries have reported high prevalence rates of depression among adolescents, for example a Saudi Arabian research reported a prevalence of depressive disorder rate of up to 42.9% [33] and in a Qatari study, depression was found to register a prevalence of 34.5% among adolescents [34]. Other studies found a much lower prevalence, for example, the prevalence rate of depression reported in Turkey was 26.6% [35] and El-Missiry [36] demonstrated that depression symptoms among Egyptian secondary school female students was approximated to be 15.3%. Ali S revealed that the prevalence rate associated with depressive symptoms among secondary school students in Dubai was 17.5% [37]. Steptoe et al. indicated that Asian countries have the highest levels of depression symptoms [38], which was consistent with the results of this study. However, the prevalence of the symptoms of depression in the present study was higher than that obtained in average people. Furthermore, other studies reported that the prevalence rate of severe adolescent depression varied from 8.7% in 2005 to 11.3% in 2014 [39]. The given difference in the results of various investigations worldwide can be due to variability in cultural factors, methodologies, instruments used for research, sampling methods, sample size, mean age differences, individuals’ motivations to answer the questionnaires, as well as lifestyles among study populations [40].

The prevalence of severe depression was high (50%), which was consistent with the results of other Iranian studies. In the study of Mohammad Zadeh et al. [41], severe depression was reported as 41%. In the study of EyvanBaga et al. [42]. Prevalence of severe depression was reported as 33% and, 52.2% of adolescents suffered from severe anxiety. In the study by Tashakori et al. [43], 82.20% of obese girls had severe depression. In studies by Daryanavard et al. [32] and Kordi et al. [44] 31.3 and 21.2% of subjects had severe depression. In explaining the probable cause of a high rate of severe depression in present study, it can be noted that adolescent students in the second high school grade were under increasing pressure to get prepared for the universities’ national entrance exam. This exam is held once each year and the acceptance rate is only about 10–15%. Therefore, participation in this highly competitive exam after high school is stressful. If they do not pass the exam, they will likely may have problems with finding the proper job in the future. Other sources of stress in adolescents are high expectations of parents from their children for admissions to the university, for a specific field of study such as medicine and engineering, and a lack of helpful counselors and supporters in schools [16]. Another possible cause might be that norms and values of sexual relationships also have been acted in Iran. Accordingly, premarital sexual relationships and emotional relationships with someone from the opposite sex are not socially accepted and are considered a disgrace to the family. However, adolescents are often hide their emotional relationships with the opposite sex, are often feel worried and guilty, have problems with sleep and concentration, feel fatigue, which are very similar to the symptoms of severe depression [45]. Nevertheless, the prevalence of severe depression in this descriptive-analytical study could be considered as a screening, which needs more clinical examinations.

### The relation between the incidence of depression and family characteristics of female students

A significant correlation was obtained between low socio-economic class and depression symptoms in female adolescence. This result may be due to the fact that present study was conducted during economic sanctions imposed against Iran when economic inequality and high risk of poverty existed; moreover, low income (below 625 dollars) and severe economic difference was observed in the majority of the study population (68.2%). Similar results were obtained by other studies [21, 46, 47]. However, the results varied from the reports of Adewuya et al. [48] and Pouretemad et al. [49]. Using different sampling methods, research methodologies, and socioeconomic classifications may be the reasons for the above-mentioned differences.

According to the results of this study, the prevalence rate of adolescent’s depression was higher in the suburbs. The higher risk of depression in the suburbs compared with that in downtown areas may be because of higher concentration of poverty and unemployment. Moreover, previous studies have indicated that residents of the suburbs had a sense of social isolation and reported lower social support [50]. These results were not consistent with the findings of other studies [22, 47] in which higher rates of mental disorders in downtown areas compared to suburb areas resulted from the faster pace of life, which can be stressful. No relationship existed between the family size and the presence of depression among female adolescence in this study which correlated with a study from New Zealand [51]. One of the possible reasons behind this result may be the higher frequency of small families compared with large ones in the present study due to population policies in Iran that had been focused on reducing the number of children in families in the previous years. Contrary to this finding, a systematic study from India reported high prevalence rate of depression in small families because a nuclear family can encounter more responsibilities without any support from other affiliated relatives [52]. On the other hand, another study reported that individuals with larger family size were more vulnerable to depression [23].

Results of this study propose that the frequency of depression was not related to the family structure type. This may be explained by the fact that divorce has been criticized by the Iranian culture. Thus, the frequency of students living only with one parent because of divorce or death was almost low (17.5%). In addition, having a child without being married is very rare in Iran. The result of the present study is consistent with the previous study [24] in which depression was not correlated with death of parents (especially mothers’ death). In this respect, various studies have suggested a relationship between parents’ status (alive/dead or living with each other/separated) and depression in adolescents [32, 53]. The reason may be that adolescents with single parent required consulting with someone about their feelings.

Parent’s occupational grade did not have protective role against female adolescent’s depression symptoms within this study.

Parent’s occupational grade did not have protective role against female adolescent’s depression symptoms within this study. This result may be due that education and occupation might be associated in a different way in the Iranian population than in the developed countries. One reason for this result may be that, although parent’s occupation, providing the economic security and high social prestige, the benefits of parent occupation and education are only internal [54].

### The relationship between incidence of depression and individual characteristics of female students

The proportion of female adolescents with depression disorder seemed to be higher in the public High Schools than in the private schools in present study. This might indicate that female students in a private high school were more likely to be endowed with greater social reinforcements and continue their education with stronger hope and motivations, which may decrease the level of anxiety or depression. These results agreed with the results of previous studies [18].

The results of this study was revealed that the fields of study were statistically and significantly correlated with depression in female students. So that students who were studying in Kar Va danesh (Job and knowledge) field of study had greater percentage of depression. One possible reason for this result can be that being academically successful and making a place for oneself in the society is Iranian adolescent’s priority and this largely depends on the field of study in high school. In today’s competitive world, it is not uncommon to find academic education as the most important role in occupational and financial status of adolescents in future [55]. In Iran’s education system, Kar Va Danesh (job and Knowledge) fields are selected due to failure in obtaining good grade point averages which may lead to dissatisfaction of students studying in these fields due to uncertainty towards future employment and social status. This result is in line with previous studies in Iran [19].

Although it has been documented in several studies [20, 56] that depression increases with age, but in the present research it was not so which may be due to age range (14–18 years of age) of the students participating in this study because they were in one secondary grade. Consistent with this, other studies [48, 57] did not confirm that depression symptoms in adolescents in higher age range was more than that in individuals of lower age range.

This study has some limitations. First, some sociodemographic questions were removed due to the legal constraints of the Iranian education system. Second these results cannot generalize other districts elsewhere in Iran because the study sample consisted of female adolescent in one county. In addition, this study cannot be generalized to the entire community because it have not a diverse sample in terms of gender. Third, in this study, parental factors did not assess what was related with adolescent depression in prior studies. Forth, the cross-sectional design of the study, because the exposure and outcome are simultaneously assessed thus causal relationships are difficult to establish. Finally, female adolescents who were not attending schools for a variety of reasons were not evaluated in this study. Subsequently, this research will only provide indications to whether specific component may or may not be possible etiological causes of depression symptoms in female students in Hamadan (west of Iran). Therefore, studies such as the case–control study with better epidemiological design are needed to elucidate causal relationships between depression in female adolescents and risk factors.

To the best of our knowledge, the present study may be the first to provide accurate information regarding depression and related factors among 14–18 year-old female adolescents in Hamadan, Iran. However, this should be regarded in the context of the methodological strengths and limitations of the study. The strengths of the present study benefits from the following: first, the sample included over 673 locally representative14–18 year-old female students and participation rate was great; second, well-performed distribution of female students (both poorest and richest areas). This study offers among female school going adolescents, an important first step into existing understanding of depressive symptoms that could become useful in developing interventions for depression in schools.

## Conclusion

This study showed a high level of depression symptoms in a sample of adolescent girls in one of the cities in western Iran. Given that 24% of adolescents in this study were screened as moderate to severe depression, it is clear that a significant number of adolescents experience mental confusion during this period, which can lead to more problems such as poor academic performance.

The high prevalence rate of depression in this study represented a growing trend in Iranian adolescents; additionally, the lower proportion of mild to moderate depression compared with severe one in the present study showed that the target population was highly exposed to environmental stressors. Therefore, the findings of the present study help clarify the socio-demographic factors influencing the mental health of female adolescents. It also provides basic knowledge for health care providers and health administrators to develop mental health policies associated with female adolescents. A periodic screening of depression in female adolescent’s population is needed to recognize those adolescents who need counseling or treatment for achieving coping skills and problem-solving abilities. Such programs can help with the improvement of coping strategies in adolescents to overcome depression problems and prevent mental health problems in this vulnerable population. For designing and implementing future preventive intervention programs, the identified factors in the current study could be helpful. Furthermore, when socio-economic factors of depression in adolescents are known, students that have these risk factors will be identified at the time of the school registration. Therefore, they can use the School Psychology Consultant. This makes it possible to intervene early and to prevent from a developed clinical disorder.

## Abbreviations

CES-D: Center for Epidemiologic Studies Depression Scale

## Footnotes

1 Kar va Danesh:Job and Knowledge; a new field of study in Iranian high schools
